# Adjunctive berberine for schizophrenia with metabolic syndrome: a systematic review and meta-analysis

**DOI:** 10.3389/fpsyt.2026.1846995

**Published:** 2026-06-24

**Authors:** Kankan Xie, Wenzhong Tian, Yang Zhang, Yan Zhang, Jianmei Li, Dong Cao, Weigang Pan

**Affiliations:** 1 Department of Psychiatry, Beijing Chaoyang Third Hospital, Beijing, China; 2 Department of Psychiatry, Chongqing Eleventh People’s Hospital, Chongqing, China; 3 Department of Psychiatry, Chongqing Jiangbei Second Hospital, Chongqing, China; 4 Department of Psychiatry, Yining People’s Hospital, Yining, Xinjiang, China; 5 Department of Psychiatry, The Affiliated Brain Hospital of Nanjing Medical University, Nanjing, China; 6 Peking University Huilongguan Clinical Medical School, Beijing Huilongguan Hospital, Capital Medical University, Beijing, China

**Keywords:** berberine, effectiveness, meta-analysis, metabolic syndrome, safety, schizophrenia

## Abstract

**Background:**

Antipsychotic-induced metabolic syndrome complicates the clinical management of schizophrenia. This investigation seeks to examine berberine’s efficacy and safety as adjunctive therapy in schizophrenia patients with metabolic syndrome.

**Methods:**

A thorough literature search was executed across international (PubMed, Cochrane Library, EMBASE) and Chinese (China National Knowledge Infrastructure, WanFang Data) databases to retrieve randomized controlled trials (RCTs) examining adjunctive berberine for schizophrenia with metabolic syndrome. Data extraction and synthesis were conducted by three independent reviewers employing RevMan 5.3 software.

**Results:**

Three eligible RCTs (n = 233) were incorporated. Adjunctive berberine demonstrated superior efficacy over controls in reducing body weight (standardized mean difference (SMD) = −0.56, I² = 0%; *P* = 0.0003), body mass index (SMD = −0.51, I² = 0%; *P* = 0.0001), waist circumference (SMD = −0.31, I² = 20%; *P* = 0.04), total cholesterol (mean difference (MD) = −0.43, I² = 63%; *P* = 0.004), triglycerides (MD = −0.31, I² = 0%; *P* < 0.0001), and fasting plasma glucose (MD = −0.30, I² = 0%; *P* = 0.005). No significant differences existed in low density lipoprotein cholesterol (MD = -0.27, I² = 75%; *P* = 0.1), high density lipoprotein cholesterol (MD = -0.02, I² = 31%; *P* = 0.56), glycated hemoglobin (MD = -0.22, I² = 67%; *P* = 0.21), systolic blood pressure (MD = -1.62, I² = 0%; *P* = 0.17), or diastolic blood pressure (MD = -1.68, I² = 39%; *P* = 0.15).

**Conclusion:**

This systematic review provides preliminary evidence supporting adjunctive berberine as a promising intervention for improving certain metabolic parameters in schizophrenia patients with metabolic syndrome. Larger, high-quality RCTs are needed to confirm these observations.

## 1 Introduction

Schizophrenia represents a chronic, debilitating mental disorder that substantially affects personal, social, and occupational functioning. Antipsychotic medications, particularly atypical antipsychotics, are the mainstay of treatment. Nevertheless, these medications link to elevated metabolic syndrome risk, featuring obesity, insulin resistance, dyslipidemia, and hypertension (1–5). A systematic review and meta-analysis indicate that the pooled global prevalence of metabolic syndrome in schizophrenia patients was 41.3% (6). This syndrome exacerbates the morbidity and mortality related to schizophrenia and its complications (7–9), posing significant challenges to the clinical management of these patients. Consequently, there is a pressing need for adjunctive treatments that can effectively address both the psychiatric and metabolic aspects of the disorder.

Current interventions for antipsychotic-induced metabolic abnormalities include lifestyle modifications (10), adjustments in antipsychotic medications (11), and targeted treatments such as metformin (12, 13). However, these approaches have notable limitations. Lifestyle changes often fail due to the vulnerability to stress and negative symptoms that are common in schizophrenia patients (14). Altering antipsychotic regimens can destabilize mental health or exacerbate psychiatric symptoms (15); metformin is endorsed by a number of guidelines for the management of antipsychotic-induced weight gain (16, 17). For example, the British Association for Psychopharmacology guidelines state that in people taking antipsychotic medications, short-term trials have shown that metformin reduces weight, compared to placebo, by approximately 3 kg (18). Indeed, a model-based meta-analysis shows its half of the maximal effect for weight control reaches 45.5 weeks, meaning short-term studies fail to capture its complete efficacy (19). However, not all patients with schizophrenia benefit from metformin (20, 21), and some patients have contraindications to metformin (22). These shortcomings highlight the need to explore alternative therapeutic options.

Berberine, an isoquinoline alkaloid from the protoberberine class, is widely used in complementary and alternative medicine for managing a variety of chronic diseases (23). Its therapeutic potential now extends beyond anti-inflammatory and gut microbiota-modulating effects to include metabolic regulation (24), which could enhance its efficacy in addressing antipsychotic-induced metabolic syndrome through synergistic actions.

Systematic reviews and meta-analyses have shown that berberine improves insulin resistance (25), lowers fasting glucose in type 2 diabetes patients (26), and reduces inflammatory markers in individuals with cardiovascular diseases, chronic kidney disease, polycystic ovary syndrome, and other comorbidities (27). A key limitation of these reviews, however, is that the studies included predominantly focused on non-psychiatric populations (25, 28, 29). Additionally, the evidence on berberine’s effects in schizophrenia patients with metabolic disturbances remains inconclusive. Some studies (30, 31) suggest that berberine beneficial effects on reducing body mass index, while others (32) report no significant impact. To date, no systematic reviews have comprehensively evaluated adjunctive berberine’s efficacy and safety in schizophrenia patients with metabolic syndrome. This review aims to fill this gap by systematically assessing the effectiveness and safety of berberine in this patient population.

## 2 Materials and methods

### 2.1 Search strategy

Three reviewers (KKX, WZT, and YZ) independently performed a comprehensive electronic literature search across international databases (PubMed, Cochrane Library, and EMBASE) and Chinese databases (China National Knowledge Infrastructure, WanFang) from database inception through December 16, 2025. Detailed search strategies are presented in **Supplementary Tables 1a****–****d**. Furthermore, the reference lists from incorporated studies (33–35) and pertinent systematic reviews along with meta-analyses (36, 37) underwent manual screening to detect any overlooked RCTs assessing adjunctive berberine’s efficacy and safety in schizophrenia patients presenting with metabolic syndrome.

### 2.2 Inclusion criteria of the meta-analysis

Per the Preferred Reporting Items for Systematic Reviews and Meta-Analyses (PRISMA) guidelines (38), inclusion criteria were developed using the PICOS framework. Participants: Patients diagnosed with schizophrenia complicated with metabolic syndrome. Schizophrenia diagnosis followed international or local criteria, including the International Classification of Diseases, 10th edition. Metabolic syndrome diagnosis followed established criteria, such as those from the International Diabetes Federation (39) or the Chinese Medical Association diabetes branch (40). Intervention (I) versus Comparison (C): Berberine combined with treatment as usual (TAU) versus placebo combined with TAU or TAU alone. Outcomes: The primary outcome encompassed reductions in four key measures: (1) anthropometric parameters (body weight, kg), body mass index (BMI, kg/m^2^), waist circumference (WC, cm); (2) serum lipid profiles (total cholesterol (TC, mmol/L), triglyceride (TG, mmol/L), high density lipoprotein cholesterol (HDL-C, mmol/L), and low density lipoprotein cholesterol (LDL-C, mmol/L)); (3) glucose metabolism markers (fasting plasma glucose (FPG, mmol/L), glycated hemoglobin (HbA1c, %)); (4) blood pressure readings (systolic blood pressure (SBP, mmHg), diastolic blood pressure (DBP, mmHg)). Secondary outcomes incorporated alterations in psychiatric symptoms, discontinuation rates, and adverse events (AEs). Study design: Only published RCTs examining adjunctive berberine’s efficacy and safety in schizophrenia patients with metabolic syndrome were incorporated. In cases of multiple publications based on the same dataset (35, 41), the study with the most comprehensive data was selected (35). Studies that lacked a clear diagnosis of metabolic syndrome (30, 31, 42–50), or those comparing adjunctive berberine with lifestyle intervention or metformin were excluded (32, 51–53).

### 2.3 Data extraction

Three reviewers (KKX, WZT, and YZ) independently retrieved the following information from the included studies: study characteristics, demographic and clinical protocol details, and outcomes. Any discrepancies between the two investigators were settled through discussion or consultation with a senior investigator (WGP). For the study with a three-arm design (berberine, metformin, and placebo) (35), only data from the berberine and placebo arms were extracted and analyzed.

### 2.4 Assessment of study quality

The quality of the included RCTs underwent independent evaluation by three reviewers (KKX, WZT, and YZ) utilizing the revised Cochrane Risk-of-Bias tool for randomized trials (ROB 2.0) (54), with each domain rated as “low risk”, “high risk”, or “some concerns” (54). This assessment with ROB 2.0 focused on the overall metabolic outcome measures.

### 2.5 Statistical analyses

Analyses were executed utilizing Review Manager Version 5.3 software, employing a random effects model (55). Only outcomes validated by two or more RCTs were incorporated in the quantitative analysis. The standardized mean differences (SMDs) or mean differences (MDs), together with their 95% confidence intervals (CIs), were computed for continuous outcomes. When both change from baseline to posttreatment values and endpoint values were available, SMD was used for pooled analysis. For all eligible studies reporting only posttreatment endpoint values, MD was adopted for data pooling. The mean and standard deviation (SD) at baseline and posttreatment were extracted from the original publications. If the SD was not reported, the 95% CI of the mean was used to impute it (33). The SD for each group was computed by dividing the CI width by 3.92 and subsequently multiplying by the square root of that group’s sample size (56):


$$\text{SD}=\sqrt{N}\times (\text{upper limit}-\text{ lower limit})/3.92$$

Study heterogeneity was evaluated utilizing the I^2^ statistic, with I^2^≥50% and *P* < 0.10 indicating significant heterogeneity (57). When I²≥50% in meta-analyses with more than two RCTs, sensitivity analysis was performed through sequential exclusion of individual outlying studies to determine the heterogeneity source. All analyses employed two-tailed testing, with statistical significance established at *P* = 0.05.

## 3 Results

### 3.1 Results of the search

**Figure 1:**
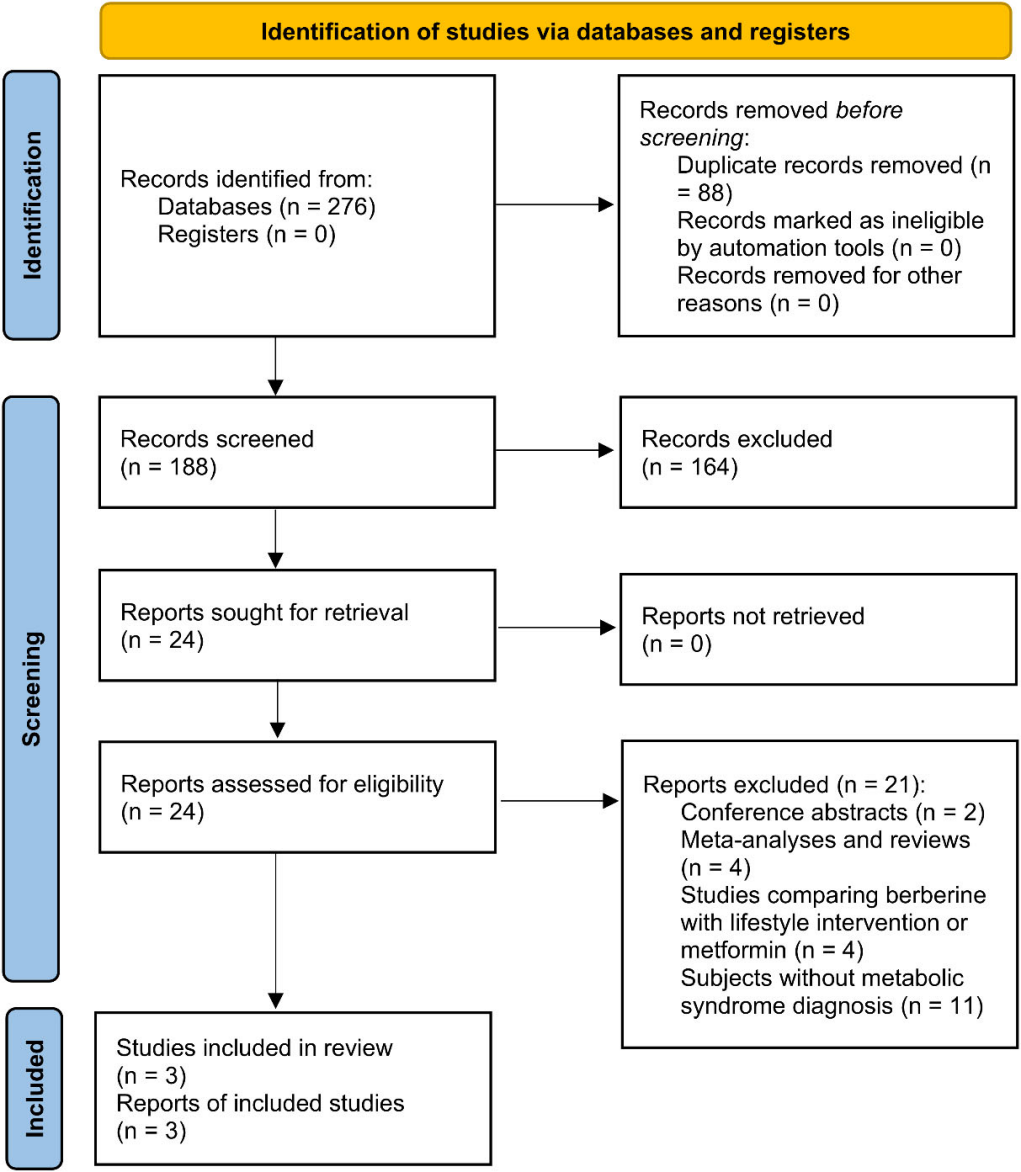
PRISMA 2020 flow diagram for systematic reviews. PRISMA, Preferred Reporting Items for Systematic reviews and Meta-Analyses.

A preliminary search retrieved 276 articles from both English (n=149) and Chinese (n=127) databases, with no additional articles identified through manual retrieval. After duplicate removal (88 studies), title and abstract screening (188 studies) and full-text assessment (24 studies), three RCTs were incorporated in this meta-analysis examining supplemental berberine impacts in schizophrenia with metabolic syndrome (32–34) (**Figure 1**).

**Table 1** presents the characteristics of the final incorporated studies. The three RCTs incorporated 233 patients with schizophrenia complicated by metabolic syndrome receiving antipsychotic treatment, with 118 patients in the berberine group and 115 in the control group. All RCTs were conducted in China. Sample sizes varied from 58 to 113 participants, with one study administering berberine at 300 mg twice daily (33), and the other two at 300 mg thrice daily (34, 35). All studies had a 12-week observation period with no follow-up beyond this duration. Two studies (32, 33, 35) used the diagnostic criteria of the International Diabetes Federation for metabolic syndrome (39), while the third study (34) adhered to the criteria of the Diabetes Branch of the Chinese Medical Association (40).

**Table 1:** Patient characteristics of each included trials.

Study (country)	Number of patients ^a^	Diagnosis (%) ^b^	Diagnostic criteria ^b^	Trial duration: (weeks)	-Illness duration: months ^b^	Mean age: yrs (range) ^b^	Sex: male (%) ^b^	Control-group dose (mg/d): mean (SD) ^c^	Intervention-group dose (mg/d): mean (SD) ^c^
Chan et al., 2022 (33) (China)	T: 113 C: 55 I: 58	SSDs ^d^ SCZ (77) SzA (8)	NR	12	129.6 (110.6)	37.8 (18-65)	49(43.4)	OLA: Ø = 13.9 (8.9)	OLA: Ø = 14.5 (8.9) BBR: Ø = 600 (FD)
Mei et al., 2016 (34) (China)	T: 58 C: 30 I: 28	SCZ (100)	ICD-10	12	NR	55.27 (18-65)	NR	APs ^f^: Ø = NR (NR)	APs ^f^: Ø = NR (NR) BBR: Ø = 900 (FD)
Zhang et al., 2023 (35) (China)	T: 62 C: 30 I: 32	SCZ (100)	ICD-10	12	208.7 (86.8) ^e^	44.47 (18-65)	41(66.1)	CPZ: Ø = 701 (190.22)	CPZ: Ø = 679.38 (136.94) BBR: Ø = 900 (FD)

Ø, mean.

^a^Data were extracted based on random assignment.

^b^Available data were extracted based on mean baseline value of each included trial.

^c^Available data were reported as means (SD).

^d^Fifteen (13.2%) patients were diagnosed with unspecific nonorganic psychosis.

Two (1.8%) patients were diagnosed with persistent delusional disorder.

^e^The original data represents the illness duration in years.

^f^Did not report the detailed use of APs.

Aps, antipsychotics; BBR, berberine; C, control; CPZ, chlorpromazine; FD, fixed dosage; I, intervention; ICD-10, International Classification of Diseases, 10th edition; NR, not reported; OLA, olanzapine; RCT, randomized controlled trial; SCZ, schizophrenia; SD, standard deviation; SSDs, schizophrenia spectrum disorders; SzA, schizoaffective disorders; T, total.

### 3.2 Risk of bias in included studies

**Figure 2:**
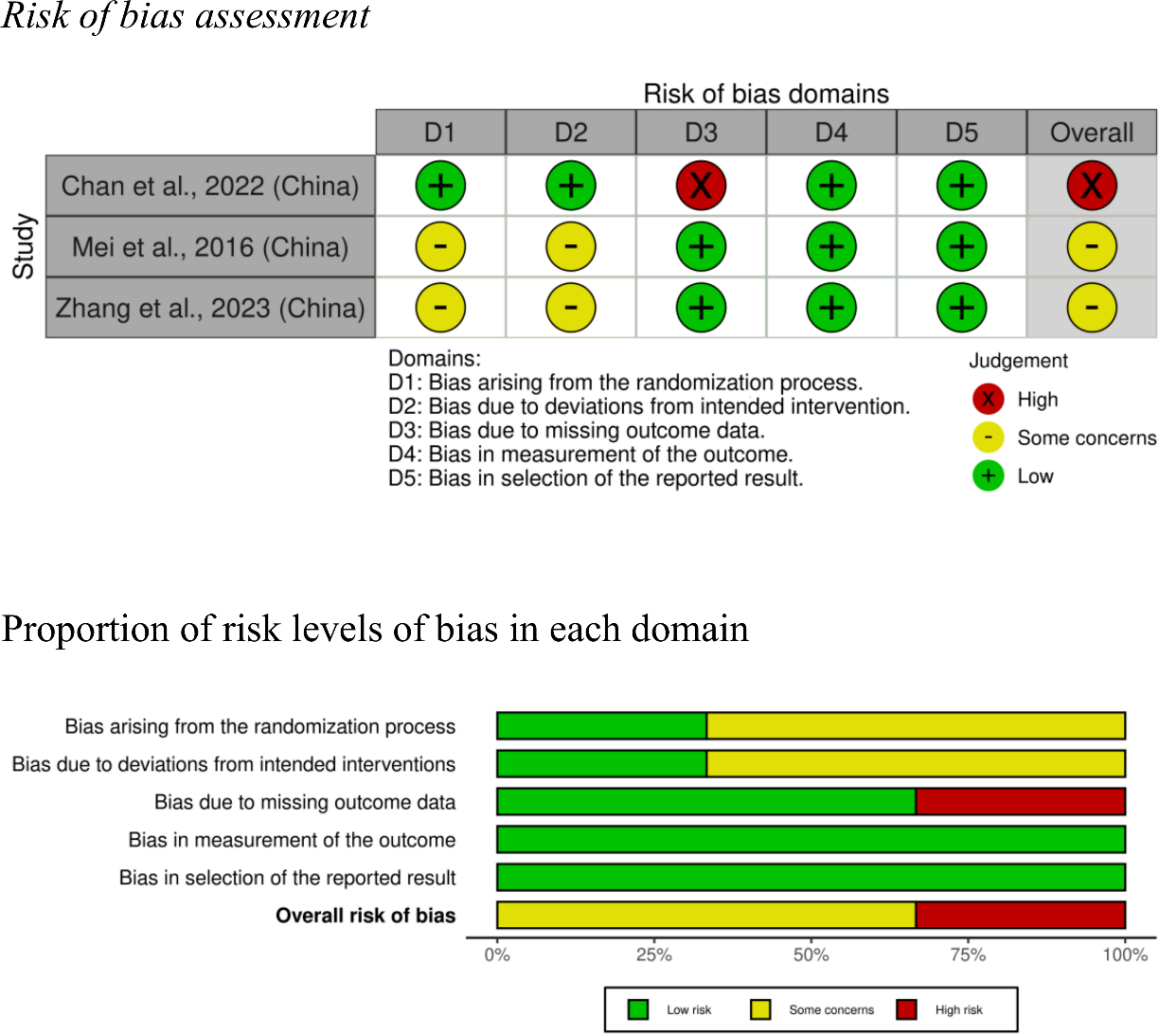
Risk of bias.

Based on the ROB 2.0 (**Figure 2**), among the three included studies, one (33) was judged as high risk and two (34, 35) raised some concerns overall. All studies (3/3, 100%) exhibited low risk of bias concerning measurement of the outcome and selection of the reported result. One study (1/3, 33.3%) (33) had low risk for randomization process and deviations from intended interventions, while the remaining two studies (34, 35) presented some concerns in these two domains. Two studies (2/3, 66.7%) (34, 35) were rated as low risk in the domain of missing outcome data, and the remaining one study (33) was rated as high risk in this domain.

### 3.3 Indicators related to metabolic syndrome

#### 3.3.1 Anthropometric parameters

**Figure 3:**
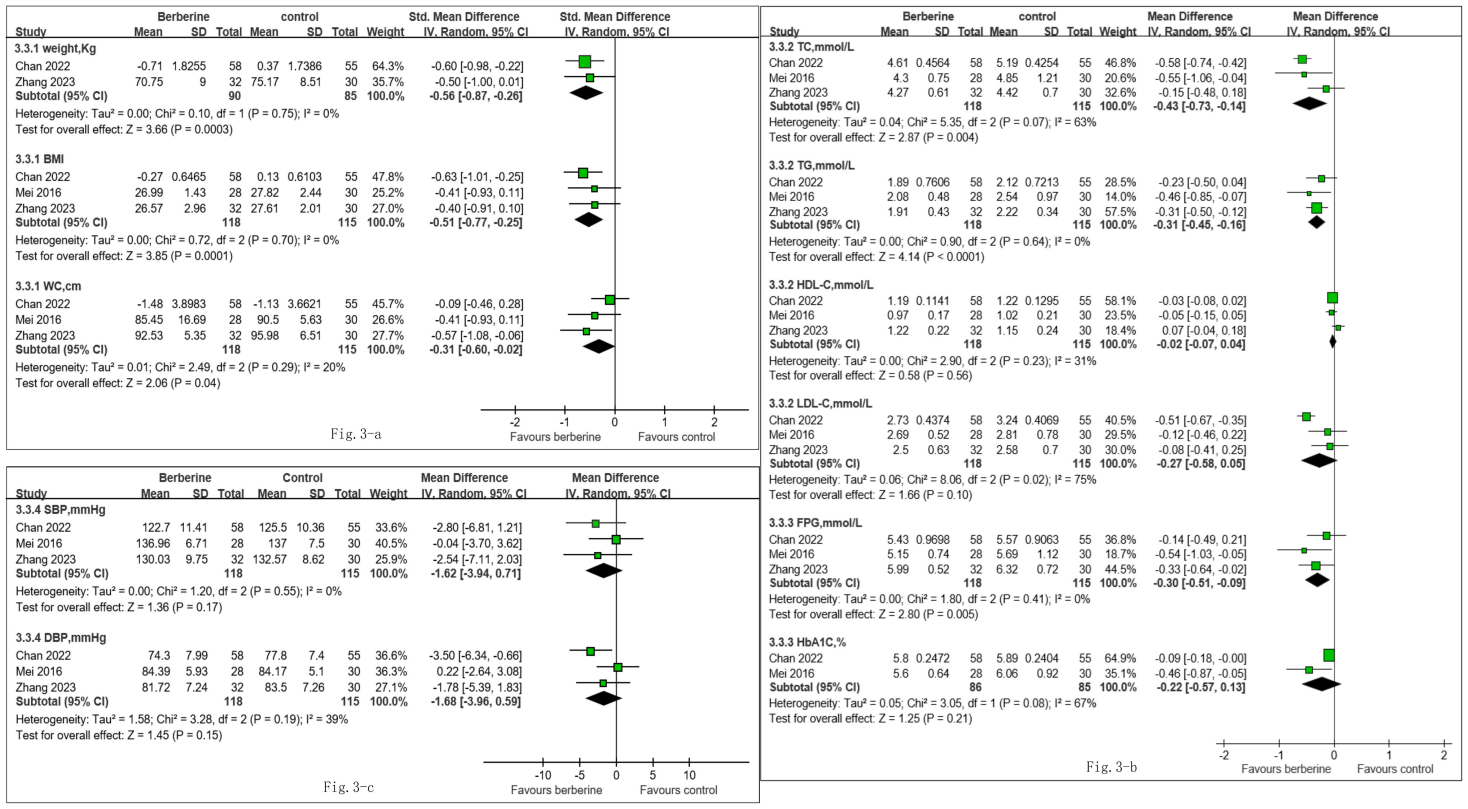
**(a)** Forest plot of anthropometric parameters for adjunctive berberine in schizophrenia patients with metabolic syndrome. **(b)** Forest plot of serum lipid and glucose metabolism markers for adjunctive berberine in schizophrenia patients with metabolic syndrome. **(c)** Forest plot of blood pressure for adjunctive berberine in schizophrenia patients with metabolic syndrome. BMI , body mass index; WC, waist circumference; SBP, systolic blood pressure; DBP, diastolic blood pressure; TC, total cholesterol; TG, triglyceride; HDL-C, high-density lipoprotein cholesterol; LDL-C, low-density lipoprotein cholesterol; FPG, fasting plasma glucose; HbA1c, glycosylated hemoglobin. For body weight, BMI and WC, Chan et al. (33) reported changes from baseline to posttreatment, while Mei (34) and Zhang (35) presented posttreatment endpoint values.

There were inconsistencies in the reporting formats of body weight, BMI and WC across included studies. One RCT (33) reported changes from baseline to posttreatment, while the other two presented posttreatment endpoint values. SD imputation was performed for studies that only reported change values. Detailed results are presented in **Supplementary Table 2**. Adjunctive berberine significantly outperformed the control group in reducing body weight (2 RCTs, SMD = −0.56, 95% CI: −0.87 to −0.26, I^2^ = 0%; *P* = 0.0003), BMI (3 RCTs, SMD = −0.51, 95% CI: −0.77 to −0.25, I^2^ = 0%; *P* = 0.0001), and WC (3 RCTs, SMD = −0.31, 95% CI: −0.60 to −0.02, I^2^ = 20%; *P* = 0.04) (**Figure 3a**).

#### 3.3.2 Serum lipid

Berberine also showed superior effects on TC level (3 RCTs, MD = −0.43, 95% CI: −0.73 to −0.14, I^2^ = 63%; *P* = 0.004), and TG level (3 RCTs, MD = −0.31, 95% CI: −0.45 to −0.16, I^2^ = 0%; *P* < 0.0001), but did not demonstrate a significant effect on HDL-C (3 RCTs, MD = −0.02, 95% CI: −0.07 to 0.04, I^2^ = 31%; *P* = 0.56) or LDL-C level (3 RCTs, MD = −0.27, 95%CI: −0.58 to 0.05, I^2^ = 75%; *P* = 0.10) (**Figure 3b**). Sensitivity analysis for LDL-C demonstrated that removing any individual RCT did not substantially alter the overall findings (*P* > 0.05). Nevertheless, regarding TC, statistical significance was markedly affected by the investigations conducted by Mei (34) and Chan (33).

#### 3.3.3 Glucose metabolism markers

Additionally, berberine showed a significant advantage over the control group in reducing FPG (3 RCTs, MD = −0.30, 95% CI: −0.51 to −0.09, I^2^ = 0%; *P* = 0.005), but no significant effect was observed on HbA1c (2 RCTs, MD = −0.22, 95% CI: −0.57 to 0.13, I^2^ = 67%, *P* = 0.21) (**Figure 3b**).

#### 3.3.4 Blood pressure

No significant differences were noted between the two groups regarding SBP (3 RCTs, MD = −1.62, 95% CI: −3.94 to 0.71, I^2^ = 0%; *P* = 0.17) and DBP (3 RCTs, MD = −1.68, 95%CI: −3.96 to 0.59, I^2^ = 39%; *P* = 0.15) (**Figure 3c**).

### 3.4 Psychiatric symptoms

Of the three RCTs that assessed adjunctive berberine for psychiatric symptoms in schizophrenia patients with metabolic syndrome, two (2/3, 66.7%) consistently showed no significant change in total psychopathology, as assessed by the Positive and Negative Syndrome Scale (PANSS), relative to control groups at study completion (32, 35). The study by Chan et al. also found no significant differences in negative, positive, or general psychopathology between the two groups (33) (**Table 2**).

**Table 2:** Adjunctive berberine and control for metabolic syndrome in patients with schizophrenia: psychiatric symptoms.

Study	Assessment scales	Findings
Berberine versus placebo control		
Chan et al., 2022 (33) (China)	PANSS	There was no significant difference in the total score of PANSS between the berberine group and the placebo group, as well as compared to baseline.
Zhang et al., 2023 (35) (China)	PANSS	There was no significant difference in the total score of PANSS between the berberine group and the placebo group, as well as compared to baseline.
Berberine versus blank control		
Mei et al., 2016 (34) (China)	NR	NR

NR, not reported; PANSS, positive and negative symptoms scale.

### 3.5 Safety assessments

Regarding safety outcomes, AEs, along with their types and frequencies, were documented in only two of the included RCTs (32, 35). One study reported 18 types of AEs (33), while the other recorded 8 (35), with details provided in **Supplementary Tables 3**, **4**. Common AEs across both studies included constipation, nausea, diarrhea, and abdominal bloating. In Chan’s study (33), drowsiness occurrence was markedly diminished in the adjunctive berberine group versus the placebo group (*P* = 0.008). Three patients withdrew due to gastrointestinal intolerance, while no significant differences in other AEs were observed between the groups. No significant differences in AEs rates were noted between the two groups in Zhang’s study (35), and notably, this study did not reported any participant withdrawals attributable to AEs.

### 3.6 Publication bias

The assessment could not be conducted due to the limited number of studies (58).

## 4 Discussion

This systematic review of three RCTs (n=233) assessed the efficacy of adjunctive berberine in managing metabolic syndrome in individuals with schizophrenia. The key findings are summarized as follows: (1) Adjunctive berberine treatment improved obesity and certain glycolipid metabolic markers, including TC, TG, and FPG, in schizophrenia patients with metabolic syndrome relative to the control group. Nevertheless, it demonstrated no significant effects on other markers such as LDL, HDL, HbA1C, and blood pressure. (2) Preliminary evidence suggests that adjunctive berberine treatment did not induce significant fluctuations in psychiatric symptoms in schizophrenia patients with metabolic syndrome. (3) Adjunctive berberine demonstrates safety and favorable tolerability within this patient population.

This systematic review explored the specific efficacy of berberine on metabolic syndrome in patients with schizophrenia, and found it exerts effects on reducing obesity and regulating glucose and lipid metabolism. Notably, multiple meta-analyses have consistently demonstrated berberine’s advantageous effects on weight reduction, lipid lowering, and glycemic control (25, 28, 29, 59, 60). For instance, Xiong et al.’s meta-analysis showed that berberine supplementation decreased BMI and WC in a dose- and time-dependent fashion among adult subjects suffering from various health conditions (60). Furthermore, Liu et al.’s review demonstrated that berberine enhanced multiple critical indicators in adults who meet at least one of the diagnostic criteria for metabolic syndrome, encompassing decreases in TG, LDL-C, TC, BMI, FPG, and WC, while exhibiting no notable impacts on HDL-C or blood pressure (SBP and DBP) (59). This systematic review builds upon these findings, further evaluating berberine’s adjunctive efficacy and safety for managing metabolic syndrome in schizophrenia, thus refining the evidence for its clinical use. The mechanisms through which berberine regulates body weight, blood lipids, and glucose are multifaceted. Animal studies and cell experiments have demonstrated that berberine inhibits adipogenesis by regulating key pathways involved in adipocyte differentiation, including peroxisome proliferator-activated receptor γ (PPARγ) and CCAAT/enhancer-binding protein α (C/EBPα) (61–63). Research executed in mice has demonstrated that berberine suppresses mitochondrial electron transport chain complex I within the gut and liver, thereby inhibiting lipid metabolism processes, encompassing intestinal fatty acid uptake and lipogenesis, along with hepatic fatty acid uptake, β-oxidation, and lipid synthesis (64, 65). In both *in vivo* and *in vitro* investigations (66, 67), berberine decreased blood glucose through modulating essential glucose metabolism mechanisms, including facilitating glycogen synthesis, suppressing gluconeogenesis, and augmenting glucose uptake. Furthermore, it mitigates insulin resistance via regulation of insulin signaling pathways, Adenosine 5’-monophosphate-activated protein kinase (AMPK)-mediated energy sensing, and epigenetic modifications. The anti-inflammatory and antioxidant characteristics of berberine also contribute to ameliorating insulin resistance in type 2 diabetes mellitus (T2DM) (67). An established interaction exists between schizophrenia and metabolic syndrome, with schizophrenia serving as a significant risk factor for metabolic syndrome development (10). Thus, the mechanisms underlying berberine-mediated improvements in metabolic syndrome in schizophrenia patients warrant further investigation.

The present meta-analysis found no significant effects of berberine on overall or specific psychotic symptoms in the included studies. However, studies involving other populations (e.g., without restriction to metabolic syndrome) suggest potential benefits of berberine for psychiatric manifestations in schizophrenia patients. A 3-month RCT suggested that berberine may alleviate negative symptoms in patients with chronic schizophrenia (42), with a reduction in the total score of the Scale for the Assessment of Negative Symptoms (SANS) in the berberine group versus the control group, and a positive correlation between changes in SANS scores and serum IL-1β levels. Another 8-week RCT (45) found significant improvements in the PANSS negative subscale scores in the berberine group. Clinical studies berberine’s anti-inflammatory properties may contribute to its beneficial effects on negative symptoms (68, 69). In contrast, among the two RCTs incorporated in the current systematic review, one investigation (33) demonstrated no significant alterations in total psychopathology, negative, positive, or general psychopathology, whereas the other investigation (35) exhibited no significant enhancement in total psychopathology. Since inflammatory cytokines are more closely linked to negative symptoms than positive symptoms (69), future studies should prioritize targeted assessments of negative symptoms, using more specific instruments such as the Clinical Assessment Interview for Negative Symptoms (CAINS) (70) and the Brief Negative Symptom Scale (BNSS) (71) to explore the therapeutic potential of berberine in this domain.

The safety of adjunctive berberine in schizophrenia patients with metabolic syndrome is supported by this systematic review. AEs were documented in only two of the included RCTs. In Chan’s study, the dropout rate was 29.3% (17/58) in the intervention group. Accordingly, we judged Domain 3 (missing outcome data) as high risk using ROB 2.0. There was no evidence that the results were not biased by missing outcome data. Additionally, regarding the reported reasons for dropout, doubts remain whether the reasons are related to efficacy or AEs (72). When the rate ratio of study discontinuation for any reason (between the two groups compared in a trial) was greater than 2 (72), such a substantial dropout rate may have important implications for the interpretation of the findings. Other studies (30, 42, 45) investigated the adjunctive use of berberine in schizophrenia patients and reported similar gastrointestinal reactions, including nausea, abdominal distension, mild abdominal pain, constipation, and diarrhea. No rare or severe AEs were observed, and most gastrointestinal reactions were mild and manageable. Based on the available evidence, schizophrenia patients with metabolic syndrome appear to tolerate adjunctive berberine well, with no serious AEs reported. Therefore, as a natural plant extract with a safety profile and low economic cost, adjunctive berberine represents a promising alternative for the long-term management of metabolic syndrome in schizophrenia patients.

This investigation presents several limitations. First, the sample sizes in the included RCTs were relatively small. Second, all participants in the included RCTs were of Chinese ethnicity, which limits the generalizability of these findings to other ethnic or regional populations. Third, one included study (34) did not report illness duration, nor did it provide detailed information on the type and dosage of antipsychotic drugs. These missing data hindered the accurate assessment of treatment efficacy and were therefore listed as a limitation of this review. Fourth, this review was not registered in a recognized international registry.

## 5 Conclusions

This meta-analysis provides preliminary evidence suggesting that adjunctive berberine has a promising yet specific metabolic profile in schizophrenia patients with metabolic syndrome, improving adiposity and certain lipids and glucose parameters, but not others (LDL-C, HDL-C, HbA1c, and blood pressure). Specifically, these findings apply only to Chinese patients with schizophrenia and metabolic syndrome aged 18–65 years, for whom adjunctive berberine (600–900 mg/day for 12 weeks) was safe and effective. The limited RCTs included, all from China, constrain the generalizability of these results, emphasizing the need for large-scale, rigorous trials to validate berberine’s efficacy and safety in this population. High-quality RCTs are needed, including those comparing different berberine dosages (600 mg/d vs 900 mg/d vs 1000 mg/d), extending follow-up to assess long-term metabolic benefits, and conducting multi-center, cross-ethnic studies to enhance external validity. Additionally, direct comparisons between berberine and metformin are essential to clarify their relative efficacy, safety, and metabolic effects.

## Data Availability

The original contributions presented in the study are included in the article/**Supplementary Material**. Further inquiries can be directed to the corresponding authors.
