# Supplementary material for: Adjunctive berberine for schizophrenia with metabolic syndrome: a systematic review and meta-analysis
Source: Front Psychiatry. 2026 Jun 24;17:1846995. doi: 10.3389/fpsyt.2026.1846995 (PMC13352474; doi:10.3389/fpsyt.2026.1846995)
Supplement: Supplementary file 1 [file Table1.docx]

**Supplemental Table 1-a. Database search strategies-PubMed**

| #1 | "Schizophrenia"[Mesh] | 121,367 |
| --- | --- | --- |
| #2 | "schizophrenia*"[Title/Abstract] OR "dementia praecox"[Title/Abstract] OR "schizophrenic disorder*"[Title/Abstract] OR "dementia precox"[Title/Abstract] OR "schizophrenic"[Title/Abstract] | 155,316 |
| #3 | "Berberine"[Mesh] | 4,958 |
| #4 | "berberine"[Title/Abstract] OR "berberin"[Title/Abstract] OR "berberine hydrochloride"[Title/Abstract] OR "berberinium chloride"[Title/Abstract] OR "umbellatine"[Title/Abstract] OR "berbines"[Title/Abstract] OR "dioxolanes"[Title/Abstract] OR "dioxoles"[Title/Abstract] OR "barberry*"[Title/Abstract] OR "berberis*"[Title/Abstract] OR "barber*"[Title/Abstract] OR "berber*"[Title/Abstract] | 11,652 |
| #5 | #1 OR #2 | 177,560 |
| #6 | #3 OR #4 | 12,252 |
| #7 | #5 AND #6 | 39 |

**Supplemental Table 1-b. Database search strategies-Embase**

| #1 | 'schizophrenia'/exp OR 'schizophrenia*':ti,ab,kw OR 'dementia praecox':ti,ab,kw OR 'schizophrenic disorder*':ti,ab,kw OR 'dementia precox':ti,ab,kw OR 'schizophrenic':ti,ab,kw | 281383 |
| --- | --- | --- |
| #2 | 'berberine'/exp OR 'berberine':ti,ab,kw OR 'berberin':ti,ab,kw OR 'berberine hydrochloride':ti,ab,kw OR 'berberinium chloride':ti,ab,kw OR 'umbellatine':ti,ab,kw OR 'berbines':ti,ab,kw OR 'dioxolanes':ti,ab,kw OR 'dioxoles':ti,ab,kw OR 'barberry*':ti,ab,kw OR 'berberis*':ti,ab,kw OR 'barber*':ti,ab,kw OR 'berber*':ti,ab,kw | 19643 |
| #3 | #1 AND #2 | 86 |

**Supplemental Table 1-c. Database search strategies-Cochrane**

| #1 | MeSH descriptor: [Schizophrenia] | 10021 |
| --- | --- | --- |
| #2 | (‘schizophrenia*’ OR ‘dementia praecox’ OR ‘schizophrenic disorder*’ OR ‘dementia precox’ OR ‘schizophrenic’): ti,ab,kw | 21379 |
| #3 | MeSH descriptor: [Berberine] | 101 |
| #4 | (‘berberine’ OR ‘berberin’ OR ‘berberine hydrochloride’ OR ‘berberinium chloride’ OR ‘umbellatine’ OR ‘berbines’ OR ‘dioxolanes’ OR ‘dioxoles’ OR ‘barberry*’ OR ‘berberis*’ OR ‘barber*’ OR ‘berber*’): ti,ab,kw | 889 |
| #5 | #1 OR #2 | 21381 |
| #6 | #3 OR #4 | 889 |
| #7 | #5 AND #6 | 24 |

**Supplemental Table 1-d. Database search strategies-Chinese database**

| **Database** | **Search strategies** | **Results** |
| --- | --- | --- |
| CNKI | (SU='小檗碱' or TI='小檗碱' or KY='小檗碱' or AB='小檗碱' or SU='小蘖碱' or TI='小蘖碱' or KY='小蘖碱' or AB='小蘖碱' or SU='盐酸小檗碱' or TI='盐酸小檗碱' or KY='盐酸小檗碱' or AB='盐酸小檗碱' or SU='黄连素' or TI='黄连素' or KY='黄连素' or AB='黄连素' or SU='盐酸黄连素' or TI='盐酸黄连素' or KY='盐酸黄连素' or AB='盐酸黄连素' or SU='BBR' or TI='BBR' or KY='BBR' or AB='BBR') and (SU='精神分裂症' or TI='精神分裂症' or KY='精神分裂症' or AB='精神分裂症' or SU='精神分裂' or TI='精神分裂' or KY='精神分裂' or AB='精神分裂' or SU='早发性痴呆' or TI='早发性痴呆' or KY='早发性痴呆' or AB='早发性痴呆') | 52 |
| Wanfang | (题名或关键词:(小檗碱 or 小蘖碱 or 盐酸小檗碱 or 黄连素 or 盐酸黄连素 or BBR) or 摘要:(小檗碱 or 小蘖碱 or 盐酸小檗碱 or 黄连素 or 盐酸黄连素 or BBR)) and (题名或关键词:(精神分裂症 or 精神分裂 or 早发性痴呆) or 摘要:( 精神分裂症 or 精神分裂 or 早发性痴呆)) | 75 |
| Abbreviations: CNKI = China National Knowledge Infrastructure. | | |

**Supplemental** **Table 2. Details of SD imputation.**

| **Study** | **Outcome** | **Mean** | **Number of Patients** | **95%CI** | **impute SD** |
| --- | --- | --- | --- | --- | --- |
| Chan et al., 2022 (China) | Change in weight | -0.71 | 58 | -1.19, -0.23 | 1.8255 |
|  | Change in BMI | -0.27 | 58 | -0.44, -0.10 | 0.6465 |
|  | Change in WC | -1.48 | 58 | -2.50, -0.45 | 3.8983 |
| Abbreviations: SD = standard deviation; CI = confidence interval; BMI = body mass index; WC = waist circumference. | | | | | |

**Supplemental Table 3.** **Adjunctive berberine and control for metabolic syndrome in patients with schizophrenia: adverse events.**

| **Study** | **Assessment scales** | **Findings** |
| --- | --- | --- |
| **Berberine versus placebo control** | | |
| Chan et al., 2022 (China) | TESS | Patients in the berberine group had significantly less incidence of drowsiness than those in the placebo group (*P* = 0.008). The incidence of other adverse events did not differ between the 2 groups. |
| Zhang et al., 2023 (China) | TESS | The main adverse reactions of berberine group are constipation and abdominal distension, and the incidence of adverse reactions is comparable to that of the control group. |
| **Berberine versus blank control** | | |
| Mei et al., 2016 (China) | NR | NR |
| Abbreviations: NR = not reported; TESS = Treatment Emergent Symptom Scale. | | |

**Supplemental Table 4.** **Adjunctive berberine and placebo for metabolic syndrome in patients with schizophrenia: discontinuation rate and adverse events.**

| **Study** | **Discontinuation rate (n, %)** | **berberine group (n, %)** | **placebo group (n, %)** | **Findings^a^** |
| --- | --- | --- | --- | --- |
| Chan et al., 2022 (China) | 25 (22.1) | 17 (29.3) | 8 (14.5) | NR |
| Mei et al., 2016 (China) | 2 (3.3) | 2 (6.7) | 0 (0) | NR |
| Zhang et al., 2023 (China) | 3 (4.6) | 1 (3.0) | 2 (6.3) | NR |
| **Study** | **Adverse effects** | **berberine group (n, %)** | **placebo group (n, %)** | **Findings^a^** |
| Chan et al., 2022 (China) | Nausea | 4 (6.9) | 3 (5.5) | NS |
|  | Vomiting | 0 (0) | 4 (7.3) | NS |
|  | Diarrhea | 8 (13.8) | 8 (14.6) | NS |
|  | Constipation | 9 (15.5) | 6 (10.9) | NS |
|  | Abdominal bloating | 5 (8.6) | 8 (14.6) | NS |
|  | Abdominal pain | 3 (5.2) | 3 (5.5) | NS |
|  | Sweating | 8 (13.8) | 5 (9.1) | NS |
|  | Tachycardia | 6 (10.3) | 7 (12.7) | NS |
|  | Dizziness | 3 (5.2) | 7 (12.7) | NS |
|  | Headache | 1 (1.7) | 6 (10.9) | NS |
|  | Insomnia | 5 (8.6) | 7 (12.7) | NS |
|  | Drowsiness | 11 (19.0) | 23 (41.8) | *P*=0.008 |
|  | Weight gain | 5 (8.6) | 4 (7.3) | NS |
|  | Attention Deficit | 9 (15.5) | 6 (10.9) | NS |
|  | Forgetfulness | 12 (20.7) | 15 (27.3) | NS |
|  | Limb stiffness | 5 (8.6) | 2 (3.6) | NS |
|  | Salivation | 8 (13.8) | 9 (16.4) | NS |
|  | Amenorrhea | 2 (3.5) | 4 (7.3) | NS |
| Mei et al., 2016 (China) | NR | NR | NR | NA |
| Zhang et al., 2023 (China) | nausea | 1 (3.1) | 1 (3.3) | NS |
|  | weakness | 2 (6.3) | 0 (0) | NS |
|  | diarrhea | 1 (3.1) | 1 (3.3) | NS |
|  | Constipation | 4 (12.5) | 1 (3.3) | NS |
|  | Abdominal bloating | 3 (9.4) | 1 (3.3) | NS |
|  | loss of appetite | 1 (3.1) | 1 (3.3) | NS |
|  | dry mouth | 1 (3.1) | 1 (3.3) | NS |
|  | Akathisia | 1 (3.1) | 2 (6.7) | NS |
| ^a^ The differences between berberine groups and control groups at the treatment endpoints.  Abbreviations: NR = not reported; NA = not applicable; NS = not significant. | | | | |
